# Association of tourniquet use on short-term implant survival after primary total knee arthroplasty: a study of 24,249 knees from the Norwegian Arthroplasty Register

**DOI:** 10.2340/17453674.2025.43981

**Published:** 2025-07-23

**Authors:** Michelle KHAN, Stein Håkon Låstad LYGRE, Mona BADAWY, Otto Schnell HUSBY, Geir HALLAN, Paul Johan HØL, Jan-Erik GJERTSEN, Ove FURNES

**Affiliations:** 1 Department of Clinical Medicine, University of Bergen, Bergen; 2 The Norwegian Arthroplasty Register, Department of Orthopaedic Surgery, Haukeland University Hospital, Bergen; 3 Department of Occupational Medicine, Haukeland University Hospital, Bergen, Norway; 4 The Coastal Hospital at Hagevik, Hagevik; 5 Department of Neuromedicine and Movement Science, Faculty of Medicine and Health Science, Norwegian University of Science and Technology, Trondheim; 6 Department of Orthopaedic Surgery, Health Møre and Romsdal HF, Kristiansund Hospital, Kristiansund; 7 Department of Orthopaedic Surgery, Biomatlab, Haukeland University Hospital, Bergen, Norway

## Abstract

**Background and purpose:**

Tourniquet use in total knee arthroplasty (TKA) provides a bloodless surgical field, which may lead to a better cementation but reduced function and increased pain. We aimed to investigate the effect of a tourniquet during TKA on implant survival, implant loosening, infection, and mortality.

**Methods:**

Data from 24,249 TKAs, collected by the Norwegian Arthroplasty Register between 2019 and 2023, was included. Among these, 14,926 were operated on with tourniquet and 9,323 without tourniquet. Cumulative revision rates (CRRs) were estimated using 1 minus Kaplan–Meier estimates for all revision causes and Cumulative Incidence Function (CIF) for specific revision causes at 3 years of follow-up. Cox regression analyses estimated hazard rate ratios (HRRs) for all revisions and Fine and Gray analyses estimated sub-hazard ratios (SHRs) for specific revision causes. Both were adjusted for age, sex, diagnosis, ASA score, fixation, implant type, and tranexamic acid use.

**Results:**

At 3 years of follow-up CRR was lower for the tourniquet group at 2.49% (95% confidence interval [CI] 2.21–2.81) vs 3.59% (CI 3.14–4.10) for the non-tourniquet group. We found an increased risk of revision in the non-tourniquet group (HRR 1.81, CI 1.46–2.46) after 3 months. CIF demonstrated a lower CRR for aseptic tibial loosening for the tourniquet group (0.08%, CI 0.04–0.15) compared with the non-tourniquet group (0.39%, CI 0.25–0.58). There was a higher risk of aseptic tibial loosening for non-tourniquet TKAs (SHR 6.06, CI 3.06–12.00), but no association with aseptic femoral loosening. There was no difference in infection or mortality.

**Conclusion:**

Tourniquet use during TKA was associated with reduced risk of tibial loosening after 3 years but without increased risk of infection, femoral loosening, or mortality.

Using a tourniquet during total knee arthroplasty (TKA) is a widely utilized practice; 59% of TKAs in Norway were performed with a tourniquet in 2022 [1], compared with 54% in Sweden and 45% in Denmark the same year [2,3]. A tourniquet is a device that restricts distal blood flow, providing a drier, bloodless field intraoperatively. This optimizes the visual conditions during surgery and may enhance implant fixation. However, inflating a tourniquet during TKA has been associated with unfortunate events such as ischemic soft tissue injury, venous thromboembolic events, increased blood loss, and increased risk for infection [4,5]. Use of a tourniquet has been shown to worsen short-term clinical outcomes, by reducing postoperative function and increasing pain [6].

Three randomized studies using radiostereometric analysis (RSA) found no difference in implant stability between TKAs performed with or without a tourniquet, with follow-up periods up to 2 years [7-9]. To our knowledge, no studies have yet been published assessing the short- or medium-term effects of tourniquet with respect to implant survival in large groups of patients from national registries. A meta-analysis by Ahmed and colleagues concluded that there is insufficient high-quality evidence to determine the direct influence of tourniquet application on implant survival. They suggested that additional research, particularly registry-based studies, may answer this question [4]. A recent systematic review by Rafaqat et al. concluded that there is a need for studies investigating the effect of tourniquet during TKA on implant survival [10].

We aimed to evaluate the short-term (3 years) association of tourniquet use in TKAs regarding implant survival. Our secondary aims included assessing revision rates for aseptic loosening, infection, and patient mortality.

## Methods

### Study design

The study from the Norwegian Arthroplasty Register (NAR) in the period 2019–2023 was performed in accordance with the STROBE statement. The surgeon fills in a form immediately after both primary and revision surgery. A revision is defined as repeat surgery in which 1 or more parts of an implant is removed, exchanged, or added [11].

### Data source

The validity and completeness of our data source, NAR, is high; completeness of reporting of procedures has been found to be 97% for primary knee replacements and 93% for revision procedures, compared with data in the Norwegian Patient Registry [1]. The report of random error in the data is found to be < 1%, where 85% of these constitute wrongful report from surgeons [12]. Furthermore, more than 95% of reoperations for periprosthetic infections within 30 days are regarded as reported, validated against the mandatory Norwegian Surveillance system for Healthcare-Associated Infections [13]. The accuracy of reported reoperations has been validated against the electronic patients records and are 87% for infections and 95% for aseptic loosening [14]. All implant components are reported using catalogue numbers using barcode scanning.

### Patients

**Figure 1:**
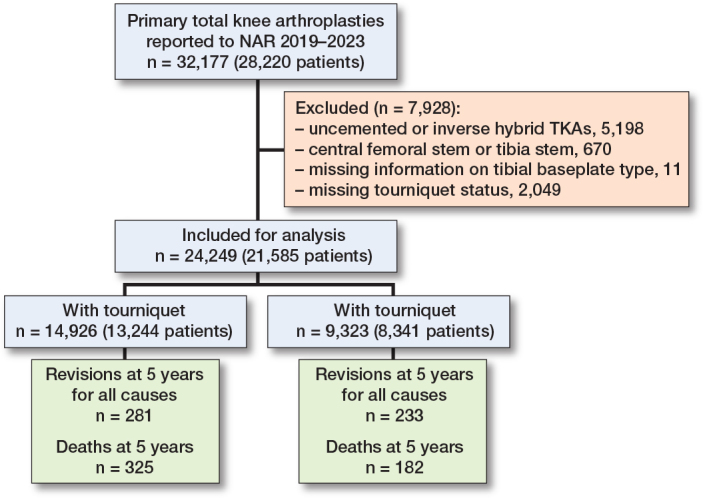
Demographic flowchart of study population.

We identified all primary TKAs performed between 2019 and 2023. Our study population included primary TKAs with either cemented or hybrid fixation (cemented tibial component). Cases not abiding by the stated criteria, or cases that lacked information on tourniquet status or tibial insert, were excluded. This selection yielded 24,249 knees eligible for analysis. Of these, 14,926 were operated on with a tourniquet and 9,323 were operated on without a tourniquet. Details concerning the case selection are described in Figure 1.

### Outcomes

We analyzed our population with respect to the following outcomes: revision of any cause as our primary endpoint, revision due to tibial loosening, femoral loosening, revision due to infection, revision due to causes other than implant loosening and infection, and patient mortality as secondary endpoints.

### Statistics

Baseline characteristics between cases operated on with and without tourniquet during surgery were compared with respect to heterogeneity using standardized mean differences. The reverse Kaplan–Meier method was used to calculate the median follow-up [15].

Crude revision rate at 3 years after primary surgery was assessed using Kaplan–Meier failure probabilities (1–KM) for all causes of revision and patient mortality. For revision, survival time started on the day of primary TKA and ended when 1 of the following events occurred: revision, emigration, death, or when the follow-up period ended (December 31, 2023), whichever came first. Bilateral knees were included in the analyses as in general this can be done without dependency issues. For analysis of mortality, if the individual had bilateral TKA the first knee was excluded, and no censoring was done when a revision took place [16]. To compare risk of revision between groups, we used Cox adjusted hazard rate ratios (HRRs) with tourniquet as the reference. For arthroplasties with multiple revision causes reported, the main cause of revision was determined based on the hierarchy from the Australian Orthopaedic Association National Joint Replacement Registry (AOANJRR) [17].

When analyzing risk of revision for specific causes (implant loosening of tibia or femur, infection, and other causes), we used Cumulative Incidence Functions (CIFs) with 3-year follow-up. Fine and Gray analyses were used to estimate sub-hazard ratios (SHRs) between the groups, with tourniquet as the reference. The 2 latter methods were used to address the issue of competing risks between specific modes of failure. For specific revision causes, the stratifying variable distinguished between no event occurring, the event of interest occurring, and a competing event occurring [18].

We developed directed acyclic graphs (DAGs) to identify confounding variables to be included in the statistical models. Our Cox and Fine and Gray models were adjusted according to this model: age (< 60 years, 60–75 years, > 75 years), sex, diagnosis (osteoarthritis [OA] or other), ASA classification (< 2 or > 3), mode of fixation (cemented or hybrid), type of tibial insert (minimally stabilized [MS], posterior stabilized [PS], rotating platform), patellar resurfacing, and tranexamic acid (used or not used) (Supplementary Figure A).

In addition, a sensitivity analysis was conducted on a subset of cases that included fully cemented TKAs with minimally stabilized (MS) tibial inserts only. Patient selection can be found in Supplementary Figure B. In the sensitivity analysis adjustment for prosthesis brand, the granularity level of the Orthopaedic Data Evaluation Panel was used [19]. The assumption of proportional hazards was evaluated statistically with Schoenfeld residuals test. To fulfill the assumption, Kaplan–Meier curves were visually inspected to determine the cut-off point at 3 months. The significance level was set at 0.05 and all tests were 2-sided.

Missing data for ASA class and tranexamic acid was assumed to be missing at random [20]. As both variables were adjusted for in Cox and Fine and Gray analysis, listwise deletion was used. To check our findings, missing imputation assigning values for “best case” (ASA class = 1–2 and receiving tranexamic acid) and “worst case” (ASA-class = 3–4 and not receiving tranexamic acid) scenarios were done (Supplementary Table 1). As an alternative to the adjusted Cox regression, we estimated the causal effect of tourniquet using an instrumental variable (IV) approach. This analysis follows the methods described by MacKenzie et al. [21] for IVs in a Cox regression model. As instrument, we applied the hospital’s annual propensity for using torniquet. Hence, the IV approach assumes that the hospital is related to risk of revision only through the use of tourniquet, and that the hospital is independent of unobserved covariates. Under these conditions the estimated HRR for tourniquet use on risk of revision can be interpreted as a causal effect estimate.

All data processing and analysis was conducted using Stata SE version 18.0 (StataCorp LLC, College Station, TX, US), apart from instrument variable analysis, which has been analyzed using the statistical package R (R Foundation for Statistical Computing, Vienna, Austria).

### Ethics, funding, use of AI tools, and disclosures

The Norwegian Data Inspectorate has granted a license to the NAR based on patient consent (reference number:16/01622-3/CDG, date of issue: latest license February 24, 2017). Collection of data was done according to Norwegian and EU data protection legislation. Data may be retrieved upon request to the NAR. The authors received no financial or material support for the research, authorship, and/or publication of this article. Chat GPT-4o was used to improve the legend beneath Supplementary Figure A. The authors declare no conflict of interest. Complete disclosure of interest forms according to ICMJE are available on the article page, doi: 10.2340/17453674.2025.43981.

## Results

Between 2019 and 2023, 32,177 primary TKAs were registered in the NAR. TKAs were excluded if they were uncemented or inverse hybrid (n = 5,198), had a central stem for either component (n = 670), lacked information on tibial baseplate type (n = 11) or lacked information on tourniquet status (n = 2,490). This selection yielded a study population of 24,249 knees, 14,926 operated on with tourniquet, and 9,323 operated on without tourniquet (Figure 1).

### Demographics

The majority (53.5%) of the TKA patients were between 60 and 75 years old, and 58.5% were female. The majority (91.3%) had OA as primary diagnosis for the TKA.

Based on standardized mean differences, there were differences in mode of fixation and patellar resurfacing between the groups, with more hybrid and more patellar resurfaced TKAs in the non-tourniquet group. Otherwise, there were small differences (Table 1).

**Table 1:** Demographic data for cases being operated with or without tourniquet during primary cemented or hybrid MS/PS/rotating platform TKA in Norway 2019–2023. Values are count (%) unless specified

Factor	Tourniquet	No	Standardized
		tourniquet	mean
	(n = 14,926)	(n = 9,323)	difference
Median follow-up (years)	2.3	1.9	
Female sex	8,649 (58)	5,538 (59)	0.030
Age, years			0.016
< 60	2,569 (17)	1,587 (17)	
60–75	8,024 (54)	4,947 (53)	
> 75	4,333 (29)	2,789 (31)	
Diagnosis			0.003
Osteoarthritis	13,619 (91)	8,514 (91)	
Other	1,307 (8.8)	809 (8.7)	
ASA class			0.036
1–2	11,458 (78)	7,059 (77)	
3	3,204 (22)	2,151 (23)	
Missing	264 (1.8)	113 (1.2)	
Tranexamic acid			0.082
Yes	14,266 (97)	8,773 (95)	
No	503 (3.4)	467 (5.1)	
Missing	157 (1.1)	83 (0.9)	
Mode of fixation			0.28
Cemented	13,172 (88)	7,243 (78)	
Hybrid**^a^**	1,754 (12)	2,080 (22)	
Type of tibial insert			0.11
Minimally stabilized**^b^**	12,005 (80)	7,526 (81)	
Posterior stabilized	1,390 (9.3)	1,404 (15)	
Rotating**^c^**	1,531 (10)	393 (4.3)	
Patellar resurfacing	1,378 (9.2)	1,623 (17)	0.24
Mean operation time,			
minutes (SD)	85.4 (21.9)	83.9 (21.9)	0.070

a Uncemented femur, cemented tibia.

b including cruciate retaining and ultra congruent polyethylene.

c Rotating platform and mobile bearing polyethylene.

Baseline characteristics for patients included in the sensitivity analysis were similar between groups apart from prosthesis brand (Supplementary Table 2).

### All-cause revision

**Figure 2:**
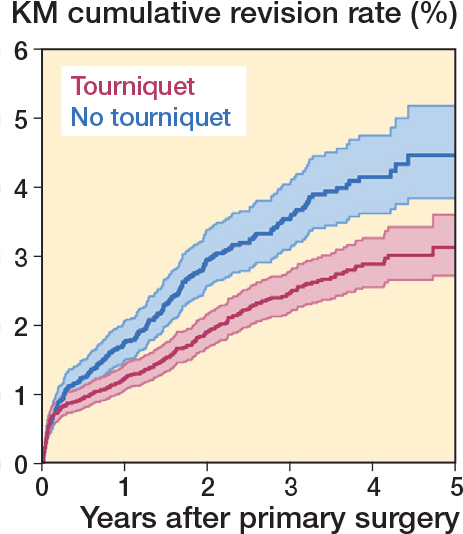
1–Kaplan–Meier cumulative all-cause revision for TKAs performed between 2019 and 2023.

At 3 years of follow-up, the 1–Kaplan–Meier probability for revision was 2.49% (CI 2.21–2.81) for cases operated on with tourniquet and 3.59% (CI 3.14–4.10) for cases operated on without. There was a statistically significant difference in risk of revision between the groups after 3 months, favoring the use of a tourniquet (HRR 1.81, CI 1.46–2.46) (Table 2, Figure 2). Instrument variable analysis on the sub-population of MS knees also showed a higher risk of all-cause revision in the non-tourniquet group after 3 months (HRR 2.86, CI 2.11–3.89) (Supplementary Table 3).

**Table 2:** Kaplan–Meier failure probabilities (1–KM) due to all causes of revision, Cumulative Incidence Function (CIF) for specific causes of revisions. Cox adjusted hazard rate ratio (HRR) for all causes and Fine and Gray adjusted sub-hazard ratios (SHR) for specific causes of revisions estimated for cases operated with or without tourniquet during primary cemented TKA in Norway 2019–2023

Time	Tourniquet	No tourniquet
	(n = 14,926)	(n = 9,323)
Revised at 3 years due to		
all causes, n	284	234
1–KM % (CI)	2.49 (2.21–2.81)	3.59 (3.14–4.10)
Patients left at risk, n	5,424	2,946
implant loosening, n	15	28
CIF% (CI)	0.15 (0.09–0.25)	0.44 (0.30–0.64)
tibial loosening, n	8	25
CIF % (CI)	0.08 (0.04–0.15)	0.39 (0.25–0.58)
femoral loosening, n	7	4
CIF % (CI)	0.07 (0.03–0.16)	0.07 (0.02–0.17)
patellar loosening, n	1	1
CIF % (CI)	0.01 (0.0–0.05)	0.03 (0.0–0.2)
infection, n	125	74
CIF % (CI)	0.95 (0.79–1.13)	0.92 (0.72–1.15)
other causes, n	144	132
CIF % (CI)	1.39 (1.17–1.64)	2.19 (1.83–2.61)
Unadjusted estimates		
HRR**^a^** all causes (CI)		
< 3 months	1	1.13 (0.86–1.48)
> 3 months	1	1.67 (1.35–2.06)
SHR**^b^** implant loosening (CI)	1	2.83 (1.60–5.00)
SHR tibial loosening (CI)	1	4.84 (2.36–9.94)
SHR femoral loosening (CI)	1	0.72 (0.23–2.29)
SHR patellar loosening (CI)	1	1.83 (0.12–29.20)
SHR infection (CI)	1	0.99 (0.75–1.32)
SHR other causes (CI)	1	1.66 (1.32–2.08)
Adjusted estimates		
HRR**^a^** all causes (CI)		
< 3 months	1	1.12 (0.84–1.49)
> 3 months	1	1.81 (1.46–2.46)
SHR**^b^** implant loosening (CI)	1	3.26 (1.80–5.89)
SHR tibial loosening (CI)	1	6.06 (3.06–12.00)
SHR femoral loosening (CI)	1	0.73 (0.19–2.76)
SHR patellar loosening (CI)	1	0.83 (0.03–19.90)
SHR infection (CI)	1	0.97 (0.73–1.29)
SHR other causes (CI)	1	1.81 (1.43–2.28)

a HRR no tourniquet vs with tourniquet (reference), estimated at 5 years of follow-up. Adjusted for age, sex, diagnosis, ASA class, tranexamic acid, mode of fixation, type of tibial insert, and patellar resurfacing. To fulfill the proportional hazards assumption, Kaplan–Meier curves were inspected and period of investigation was split at 3 months. Simultaneous loosening: 1 and 2 knees were revised with simultaneous femoral and tibial loosening in the tourniquet and no tourniquet group respectively .

b Cumulative SHR no tourniquet vs tourniquet (reference) estimated at 5 years of follow-up. Adjusted for age, sex, diagnosis, ASA class, tranexamic acid, mode of fixation, type of tibial insert, and patellar resurfacing.

### Aseptic implant loosening

**Figure 3:**
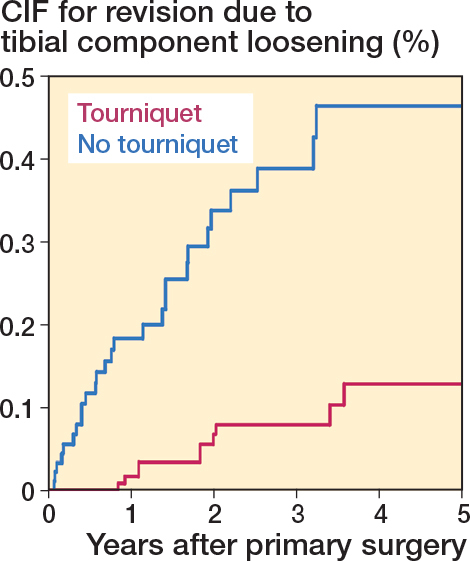
Cumulative incidence function (CIF) for revision due to aseptic tibial loosening for TKAs performed between 2019 and 2023.

**Figure 4:**
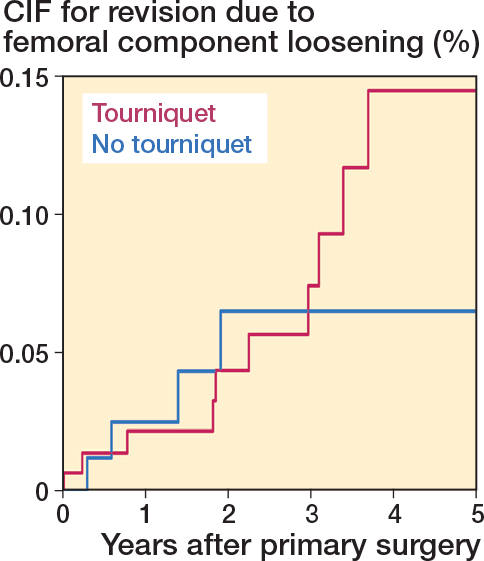
Cumulative incidence function (CIF) for revision due to aseptic femoral loosening for TKAs performed between 2019 and 2023.

At 3 years of follow-up, CIF showed higher rates for tibial loosening 0.39% (CI 0.25–0.58) in the non-tourniquet group compared with 0.08% (CI 0.04–0.15) in the tourniquet group (SHR 6.06 CI 3.06–12.00) (Table 2, Figure 3). There was no difference in CIF for femoral loosening 0.07 (CI 0.03–0.16) vs 0.07 (CI 0.02–0.17) for tourniquet and non-tourniquet respectively (SHR 0.73, CI 0.19–2.76) (Table 2, Figure 4).

### Infection

**Figure 5:**
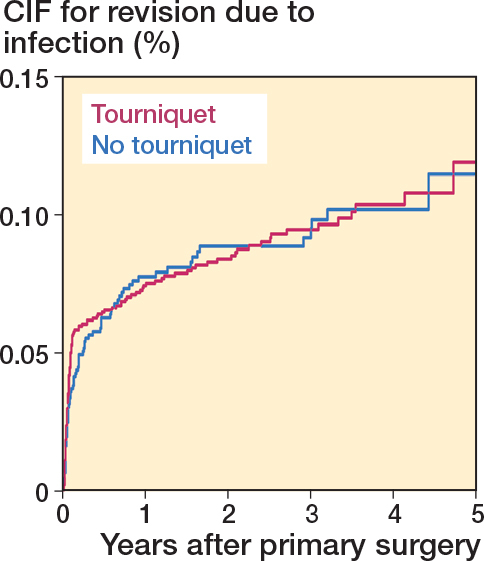
Cumulative incidence function (CIF) for revision due to infection for TKAs performed between 2019 and 2023.

At 3 years of follow-up, CIFs showed similar rates of revision for infection; 0.95% (CI 0.79–1.13) for patients operated on with tourniquet and 0.92% (CI 0.72–1.15) for patients operated on without (Table 2, Figure 5). Adjusted Fine and Gray analysis did not show a statistically significant difference in risk of infection (Table 2).

### Other revision causes

For other revision causes than infection and implant loosening, CIFs showed lower rates of revision, 1.39% (CI 1.17–1.64) for TKAs with tourniquet, than for TKAs without, 2.19% (CI 1.83–2.61). Fine and Gray analysis revealed a statistically significant increased risk of revision by other causes for knees operated on without tourniquet (Table 2). Specific revision causes are summarized in Supplementary data. There was an increased risk of periprosthetic fracture for knees operated on without a tourniquet (Supplementary Table 4).

### Mortality

**Figure 6:**
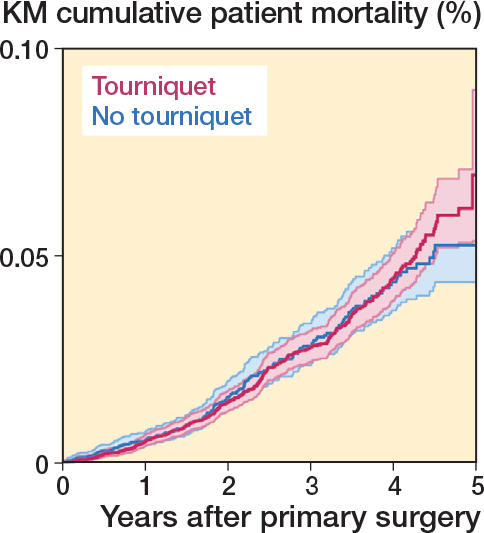
1–Kaplan–Meier cumulative mortality for patients operated on between 2019 and 2023.

At 1 year of follow-up, the cumulative mortality risk analysis indicated that the mortality probabilities for patients operated on with and without a tourniquet were similar, at 0.54% (CI 0.42–0.69) and 0.55% (CI 0.40–0.76) respectively (Table 3, Figure 6). There was no statistically significant difference in the adjusted HRR, 0.95 (CI 0.79–1.16) (Table 3).

**Table 3:** Kaplan–Meier cumulative mortality rate for patients operated on with or without tourniquet during their primary TKA in Norway between 2019 and 2023. Cox adjusted hazard rate ratio for risk of death

Time	Tourniquet	No tourniquet
	(n = 13,244)	(n = 8,341)
Dead at 30 days, n	4	5
1–KM % (CI)	0.03 (0.01–0.08)	0.06 (0.03–0.15)
Patients left at risk, n	13,004	8,135
Dead at 1 year, n	62	39
1–KM % (CI)	0.54 (0.42–0.69)	0.55 (0.40–0.76)
Patients left at risk, n	10,170	5,559
Dead at 3 years, n	224	127
1–KM % (CI)	2.80 (2.45–3.20)	2.82 (2.36–3.38)
Patients left at risk, n	4,701	2,580
Unadjusted estimates		
Hazard rate ratio **^a^** (CI)	1	0.98 (0.81–1.19)
Adjusted estimates		
Hazard rate ratio **^b^** (CI)	1	0.95 (0.79–1.16)

a No tourniquet vs with tourniquet as reference.

b Adjusted for age, sex, diagnosis, ASA class, tranexamic acid.

### Sensitivity analysis and missing imputation analysis

The missing imputation analysis for best and worst case scenarios for the whole study population yielded similar results. The sensitivity analysis and imputation analysis, including fully cemented TKAs with MS tibial inserts only, yielded comparable results to the total study population (Supplementary Tables 2, 3, 5, 6 and Figure A and B).

## Discussion

We aimed to investigate the effect of a tourniquet during TKA on implant survival, implant loosening, infection, and mortality. We found that refraining from using a tourniquet during TKA was associated with increased risk of all-cause revision with 5 years of follow-up. When looking at specific causes of revision, omitting a tourniquet was associated with an increased risk of revision due to aseptic tibial loosening and of revision due to other causes. Our data showed that the femoral component was not as susceptible to aseptic loosening as its tibial counterpart, and that tourniquet use did not affect the risk of revision for femoral component loosening. Risk of revision due to infection and mortality was not affected by tourniquet use.

### Aseptic tibial loosening

In this study, revision for early aseptic tibial loosening occurred less frequently when using a tourniquet during surgery. A retrieval study by Miller et.al. found that increased cement penetration increased the stability of the tibial baseplate [22]. It has been suggested that aiming for tibial cement penetration of between 3 and 5 mm is advisable in order to increase implant stability [23]. Preventing contamination, e.g., by blood, of the tibial baseplate has a protective effect on tibial fixation [24]. The effect of a tourniquet on cement penetration has been investigated, with conflicting results. Both a meta-analysis [6] and an RCT conducted by Peker and Altun [25] demonstrated that using a tourniquet increased the cement penetration significantly. Conversely, a scoping review assessing 16 RCTs [10] and a meta-analysis reviewing a total of 41 RCTs [4,26] concluded that there was no improvement in implant stability when inflating a tourniquet. However, most of the studies had a maximum follow-up of only 2 years and were small, ranging from 20–103 knees. A retrospective comparative clinical study found TKAs with tourniquet to be more stable after 3 years’ follow-up [27], which is supported by our findings.

### Infection, death, and venous thromboembolic events

We found no indication that a tourniquet influenced the risk of revision due to infection. Our finding contrasted with a meta-analysis on 14 RCT studies conducted by Magan et al. that found an increased risk of infection when using a tourniquet during surgery [5]. In addition, Ahmed et al. conducted a meta-analysis assessing the risk for infection with similar conclusions [4,26]. Apart from these studies, there have, to our knowledge, been no registry studies assessing the effect of tourniquet on risk of deep infection. The ability of arthroplasty registers to accurately evaluate infection rates has been questioned [14,28], and the true incidence of infection is presumably underestimated. However, there is no reason to believe that this would unequally affect the study groups in the present study.

We assessed mortality as a proxy for serious thromboembolic events and could not find a difference between the groups. Magan and colleagues found no increased risk of VTE and postoperative drop in hemoglobin levels when applying a tourniquet [5]. 2 Danish multi-center prospective cohort studies yielded similar results [29,30]. In contrast, a meta-analysis by Liu and colleagues found a significantly increased risk of VTE when using a tourniquet, especially for longer periods of time [31]. Our data was limited to mortality, and less serious VTEs were not studied. Thus there is a chance that the use of a tourniquet may affect the risk of non-fatal VTE despite our finding that the mortality was the same in the 2 groups.

Proponents of omitting the tourniquet argue that recovery is faster and the risk of VTE smaller without a tourniquet [30]. However, multiple studies show that there is no difference in functional scores or pain 6 months postoperatively [32-34]. In the present study of cemented/hybrid TKAs we found a higher risk of implant revision in the non-tourniquet group (3.6% vs 2.5%). Revision for tibial component loosening was the main reason for this difference. The higher risk of tibial loosening could be caused by suboptimal cementing on a bloody tibial cut surface. We found no difference in mortality or infection rates, indicating that the short tourniquet time (average < 85 minutes) does not cause higher risk of death due to thromboembolic disease or higher risk of periprosthetic infection. As long as antibiotic prophylaxis is given more than 30 minutes before tourniquet application the tissue should have a high enough concentration of antibiotic to prevent infection. Thus our findings support the use of a tourniquet. A slightly slower recovery could be justified by a reduced risk of revision surgery. Another approach could be to apply a tourniquet only during cementation, to get the benefits of a dry, bloodless surgical field, as well as reduce risks of complications associated with long-term occlusion.

### Strengths

The major strength of this study is the high number of TKAs included, which is higher than any other study on this topic. The NAR collects data with standardized forms from all Norwegian hospitals with high coverage and completeness of both primary and revision cases. In addition, our study provides similar or longer follow-up than most other studies on the topic with a median follow-up of 2.3 years for knees operated on with a tourniquet and 1.88 years for knees operated on without a tourniquet. Maximum follow-up was 5 years.

### Limitations

Our study did not have patient-reported outcomes. Therefore we could not study functional recovery. In addition, the relatively small number of revisions due to specific causes must be considered when evaluating the findings, as well as the wide confidence intervals for the Fine and Gray and Cox analyses. We are aware of BMI, activity level, and surgeon as potential confounders; however, we were unable to control for these factors as the NAR did not collect BMI data during the study period and does not collect data on activity level or surgeon. The standard in Norway has been using a tourniquet, and surgeons most likely to swap technique could be younger or may be more dedicated to knee replacement surgery. Also it is possible that in complex cases (stiff or malaligned knees, high BMI) a tourniquet was used more frequently. These factors could skew our results, but we find it unlikely that the effect would be significant. Regarding the difference in aseptic tibial loosening, it is impossible to rule out residual confounding. We conducted an instrument variable analysis, using hospital and year of operation as instruments to account for such confounding, revealing similar conclusions. We did not assess blood loss in the current study, but the issue is of importance in TKA. There are several meta-analyses concluding that inflating a tourniquet does not increase total blood loss [4,35].

### Conclusion

The use of a tourniquet was associated with reduced risk of aseptic tibial loosening after 3 years but not with risk of infection, femoral loosening, or mortality.

Tourniquet use improved implant survival, especially by being associated with a reduced risk of revision due to aseptic tibial loosening. The risk of revision due to prosthetic joint infection was not affected by tourniquet use during TKA and neither was mortality.

*In perspective,* more registry studies with longer follow-up confirming our data, preferably with PROM data alongside implant survival data, would be valuable.

### Supplementary data

Supplementary Tables (1–6) and Figures (A and B) are available as Supplementary data on the article page, doi: 10.2340/17453674.2025.43981

## Supplementary Material



## Notes

MK: statistical analysis, drafting the first and last version of manuscript. OF, J-EG: creation of study design and protocol, review of manuscript. SHLL: review and advice on statistical analysis, review of manuscript. PJH, GH, MB, OSH: review of manuscript.

The authors are grateful to the orthopedic surgeons in Norway who continuously report to the NAR, and to the NAR providing the necessary data for analysis to this study.

Handling co-editors: Li Felländer-Tsai and Robin Christensen

*Acta* thanks Harald Brismar and Anders El-Galaly for help with peer review of this manuscript.

## References

[1] Furnes O, Gjertsen J-E, Hallan G, Visnes H, Gundersen T, Fenstad A M, et al. Årsrapporten 2022. Helse Bergen HF; 2023. https://www.helse-bergen.no/nasjonalt-kvalitets-og-kompetansenettverk-for-leddproteser-og-hoftebrudd/arsrapporter/#annual-reports

[2] W-Dahl A, Rogmark C, Johannson O, Arani P A, Mohaddes M, Rolfson O. Svenska Ledprotesregistrets årsrapport 2024 med data för 2023; 2024. doi: 10.18158/i_2vVZosV. https://slr.registercentrum.se/nyheter/ledprotesregistrets-arsrapport-2024-finns-nu-att-ladda-ner

[3] Lindberg-Larsen M. Dansk Knæalloplastik Register Årsrapport 2022; 2023. https://sundk.dk/kliniske-kvalitetsdatabaser/dansk-knaealloplastik-register-dkr/viden-fra-databasen/429331bd55fd4176a75ccdb6be73e5b6.pdf

[4] Ahmed I, Chawla A, Underwood M, Price A J, Metcalfe A, Hutchinson C E, et al. Time to reconsider the routine use of tourniquets in total knee arthroplasty surgery. Bone Joint J 2021; 103-B(5): 830-9. doi: 10.1302/0301-620X.103B.BJJ-2020-1926.R1 33683139 PMC8091001

[5] Magan A A, Dunseath O, Armonis P, Fontalis A, Kayani B, Haddad F S. Tourniquet use in total knee arthroplasty and the risk of infection: a meta-analysis of randomised controlled trials. J Exp Orthop 2022; 9(1): 62. doi: 10.1186/s40634-022-00485-9. 35776268 PMC9249956

[6] Han J, Zhang X Y, Mu S Y, Liu S L, Cui Q T, Zhang C, et al. Tourniquet application in primary total knee arthroplasty for osteoarthritis: a systematic review and meta-analysis of randomized controlled trials. Front Surg 2022; 9: 994795. doi: 10.3389/fsurg.2022.994795. 36684363 PMC9852050

[7] Molt M, Harsten A, Toksvig-Larsen S. The effect of tourniquet use on fixation quality in cemented total knee arthroplasty: a prospective randomized clinical controlled RSA trial. Knee 2014; 21(2): 396-401. doi: 10.1016/j.knee.2013.10.008. 24238650

[8] Ejaz A, Laursen A C, Jakobsen T, Rasmussen S, Nielsen P T, Laursen M B. Absence of a tourniquet does not affect fixation of cemented TKA: a randomized RSA study of 70 patients. J Arthroplasty 2015; 30(12): 2128-32. doi: 10.1016/j.arth.2015.05.058. 26162514

[9] Ledin H, Aspenberg P, Good L. Tourniquet use in total knee replacement does not improve fixation, but appears to reduce final range of motion. Acta Orthop 2012; 83(5): 499-503. doi: 10.3109/17453674.2012.727078. 22974220 PMC3488177

[10] Rafaqat W, Kumar S, Ahmad T, Qarnain Z, Khan K S, Lakdawala R H. The mid-term and long-term effects of tourniquet use in total knee arthroplasty: systematic review. J Exp Orthop 2022; 9(1): 42. doi: 10.1186/s40634-022-00471-1. 35552912 PMC9098769

[11] Havelin L I, Engesaeter L B, Espehaug B, Furnes O, Lie S A, Vollset S E. The Norwegian Arthroplasty Register: 11 years and 73,000 arthroplasties. Acta Orthop Scand 2000; 71(4): 337-53. doi: 10.1080/000164700317393321. 11028881

[12] Arthursson A J, Furnes O, Espehaug B, Havelin L I, Soreide J A. Validation of data in the Norwegian Arthroplasty Register and the Norwegian Patient Register: 5,134 primary total hip arthroplasties and revisions operated at a single hospital between 1987 and 2003. Acta Orthop 2005; 76(6): 823-8. doi: 10.1080/17453670510045435. 16470436

[13] Karlsen O E, Dale H, Furnes O, Eriksen-Volle H M, Westberg M. Trends in surgical site infection and periprosthetic joint infection after primary total hip arthroplasty in two national health registers 2013-2022. J Hosp Infect 2025; 159: 148-55. doi: 10.1016/j.jhin.2025.01.010. 39924115

[14] Lutro O, Mo S, Tjorhom M B, Fenstad A M, Leta T H, Bruun T, et al. How good are surgeons at disclosing periprosthetic joint infection at the time of revision, based on pre- and intra-operative assessment? A study on 16,922 primary total hip arthroplasties reported to the Norwegian Arthroplasty Register. Acta Orthop 2024; 95: 67-72. doi: 10.2340/17453674.2024.39914. 38288989 PMC10826841

[15] Schemper M, Smith T L. A note on quantifying follow-up in studies of failure time. Control Clin Trials 1996; 17(4): 343-6. doi: 10.1016/0197-2456(96)00075-x. 8889347

[16] Robertsson O, Ranstam J. No bias of ignored bilaterality when analysing the revision risk of knee prostheses: analysis of a population based sample of 44,590 patients with 55,298 knee prostheses from the national Swedish Knee Arthroplasty Register. BMC Musculoskelet Disord 2003; 4: 1. doi: 10.1186/1471-2474-4-1. 12570876 PMC150595

[17] Smith P N, Gill D R, McAuliffe M J, McDougall C, Stoney J D, Vertullo C J, et al. Hip, Knee and Shoulder Arthroplasty: 2023 Annual Report, Australian Orthopaedic Association National Joint Replacement Registry. Adelaide, South Australia: AOA; 2023. 10.25310/YWQZ9375.

[18] Lie S A, Fenstad A M, Lygre S H L, Kroken G, Dybvik E, Gjertsen J E, et al. Kaplan–Meier and Cox regression are preferable for the analysis of time to revision of joint arthroplasty: thirty-one years of follow-up for cemented and uncemented THAs inserted from 1987 to 2000 in the Norwegian Arthroplasty Register. JB JS Open Access 2022; 7(1) 1. doi: 10.2106/jbjs.Oa.21.00108. PMC8865509 35224411

[19] ODEP. Orthopaedic Data Evaluation Panel 2025. Available from: https://www.odep.org.uk/about/introducing-odep/

[20] Christensen R, Ranstam J, Overgaard S, Wagner P. Guidelines for a structured manuscript: statistical methods and reporting in biomedical research journals. Acta Orthop 2023; 94: 243-9 1. doi: 10.2340/17453674.2023.11656. 37170796 PMC10176201

[21] MacKenzie T A, Tosteson T D, Morden N E, Stukel T A, O’Malley A J. Using instrumental variables to estimate a Cox’s proportional hazards regression subject to additive confounding. Health Serv Outcomes Res Methodol 2014; 14(1-2): 54-68. doi: 10.1007/s10742-014-0117-x. 25506259 PMC4261749

[22] Miller M A, Terbush M J, Goodheart J R, Izant T H, Mann K A. Increased initial cement–bone interlock correlates with reduced total knee arthroplasty micro-motion following in vivo service. J Biomech 2014; 47(10): 2460-6. doi: 10.1016/j.jbiomech.2014.04.016. 24795171 PMC4100248

[23] Refsum A M, Nguyen U V, Gjertsen J E, Espehaug B, Fenstad A M, Lein R K, et al. Cementing technique for primary knee arthroplasty: a scoping review. Acta Orthop 2019; 90(6): 582-9 1. doi: 10.1080/17453674.2019.1657333. 31452416 PMC6844414

[24] Silva M M B, Gjertsen J E, Moldestad I O, Furnes O N, Khan M, Hol P J. Effects of implant precoating and fat contamination on the stability of the tibial baseplate. Knee 2024; 49: 266-78 1. doi: 10.1016/j.knee.2024.07.007. 39059126

[25] Peker G, Altun I. Total knee arthroplasty: the impact of tourniquet usage on cement penetration, operation time, and bleeding control. Med Sci Discovery 2023. 10.36472/msd.v10i9.1035.

[26] Ahmed I, Chawla A, Underwood M, Price AJ, Metcalfe A, Hutchinson C, et al. Tourniquet use for knee replacement surgery. Cochrane Database Syst Rev 2020; 12(12): Cd012874. doi: 10.1002/14651858.CD012874.pub2. 33316105 PMC8094224

[27] Touzopoulos P, Ververidis A, Mpogiatzis C, Chatzigiannakis A, Drosos G I. The use of tourniquet may influence the cement mantle thickness under the tibial implant during total knee arthroplasty. Eur J Orthop Surg Traumatol 2019; 29(4): 869-75. doi: 10.1007/s00590-019-02369-8. 30617921

[28] Gundtoft P H, Overgaard S, Schonheyder H C, Moller J K, Kjaersgaard-Andersen P, Pedersen A B. The “true” incidence of surgically treated deep prosthetic joint infection after 32,896 primary total hip arthroplasties: a prospective cohort study. Acta Orthop 2015; 86(3): 326-34. doi: 10.3109/17453674.2015.1011983. 25637247 PMC4443464

[29] Petersen P B, Mikkelsen M, Jorgensen C C, Kappel A, Troelsen A, Kehlet H, et al. Use of a tourniquet is not associated with increased risk of venous thromboembolism after fast-track total knee arthroplasty: a prospective multicenter cohort study of 16,250 procedures. Acta Orthop 2023; 94: 342-7. doi: 10.2340/17453674.2023.13793. 37417882 PMC10327675

[30] El-Galaly A, Hansen A T, Kappel A. The use of tourniquet in primary total knee arthroplasty does not increase the risk of venous thromboembolism within 90 days of surgery: a Danish nationwide cohort study of 19,804 patients. Knee Surg Sports Traumatol Arthrosc 2023; 31(3): 883-91. doi: 10.1007/s00167-022-06965-w. 35445851

[31] Liu Y, Si H, Zeng Y, Li M, Xie H, Shen B. More pain and slower functional recovery when a tourniquet is used during total knee arthroplasty. Knee Surg Sports Traumatol Arthrosc 2020; 28(6): 1842-60. doi: 10.1007/s00167-019-05617-w. 31289914

[32] Liu P L, Li D Q, Zhang Y K, Lu Q S, Ma L, Bao X Z, et al. Effects of unilateral tourniquet used in patients undergoing simultaneous bilateral total knee arthroplasty. Orthop Surg 2017; 9(2): 180-5. doi: 10.1111/os.12329. 28598560 PMC6584465

[33] Zhou K, Ling T, Wang H, Zhou Z, Shen B, Yang J, et al. Influence of tourniquet use in primary total knee arthroplasty with drainage: a prospective randomised controlled trial. J Orthop Surg Res 2017; 12(1): 172. doi: 10.1186/s13018-017-0683-z. 29137681 PMC5686948

[34] Goel R, Rondon A J, Sydnor K, Blevins K, O’Malley M, Purtill J J, et al. Tourniquet use does not affect functional outcomes or pain after total knee arthroplasty: a prospective, double-blinded, randomized controlled trial. J Bone Joint Surg Am 2019; 101(20): 1821-8. doi: 10.2106/JBJS.19.00146. 31626006

[35] Li X, Yin L, Chen Z Y, Zhu L, Wang H L, Chen W, et al. The effect of tourniquet use in total knee arthroplasty: grading the evidence through an updated meta-analysis of randomized, controlled trials. Eur J Orthop Surg Traumatol 2014; 24(6): 973-86. doi: 10.1007/s00590-013-1278-y. 23842662

