# Supplementary material for: Association of tourniquet use on short-term implant survival after primary total knee arthroplasty: a study of 24,249 knees from the Norwegian Arthroplasty Register
Source: Acta Orthop. 2025 Jul 23;96:547–54. doi: 10.2340/17453674.2025.43981 (PMC12285508; doi:10.2340/17453674.2025.43981)
Supplement: Supplementary file 1 [file ActaO-96-43981-s1.pdf]

Supplementary data

Tables

Table 1. Missing imputation with “best case” and “worst case” scenarios for tranexamic acid and ASA class on cases being operated on with or without tourniquet during primary cemented or hybrid MS/PS/rotating platform TKA with non-resurfaced and resurfaced patella components in Norway 2019–2023

	Tourniquet (reference)	No tourniquet
“Best case” [a] imputation		
HRR [c] all causes (CI)		
< 3 months	1	1.08 (0.82–1.42)
> 3 months	1	1.66 (1.34–2.05)
SHR [d] implant loosening (CI)	1	3.26 (1.81–5.90)
SHR tibial loosening (CI)	1	6.06 (3.06–12.0)
SHR femoral loosening (CI)	1	0.73 (0.20–2.77)
SHR patellar loosening (CI)	1	0.87 (0.04–17.80)
SHR infection (CI)	1	0.96 (0.72–1.27)
SHR other causes (CI)	1	1.84 (1.46–2.32)
“Worst case” [b] imputation		
HRR all causes (CI)		
< 3 months	1	1.09 (0.82–1.43)
> 3 months	1	1.67 (1.35–2.07)
SHR implant loosening (CI)	1	3.29 (1.82–5.93)
SHR tibial loosening (CI)	1	6.11 (3.09–12.10)
SHR femoral loosening (CI)	1	0.73 (0.19–2.77)
SHR patellar loosening (CI)	1	0.83 (0.03–19.90)
SHR infection (CI)	1	0.96 (0.72–1.28)
SHR other causes (CI)	1	1.85 (1.47–2.33)

[a] Best case: Tranexamic acid and ASA class 1–2.
[b] Worst case: No tranexamic acid and ASA class 3–4.
[c] Hazard rate ratio (HRR) for no tourniquet vs with tourniquet (reference) adjusted for age, sex, diagnosis, ASA class, tranexamic acid, mode of fixation, type of tibial insert, and patellar resurfacing. To fulfill the proportional hazards assumption Kaplan-Meier curves were inspected and period of investigation were split at 3 months.
[d] Sub-hazard ratio (SHR) for no tourniquet vs with tourniquet (reference) adjusted for age, sex, diagnosis, ASA-class, tranexamic acid, mode of fixation, type of tibial insert, and patellar resurfacing.

Table 2. Sensitivity analysis. Demographic data for cases being operated on with or without tourniquet during primary cemented CR/UC[a] TKA with both non-resurfaced and resurfaced patella in Norway 2019–2023. Values are count (%) unless otherwise specified

Factor	Tourniquet (n = 10,334)	No tourniquet (n = 5,622)	Standardized mean difference
Median follow up (years) [b]	2.25	1.70	
Female sex	5,997 (58)	3,284 (58)	0.0077
Age			
< 60 years	1,838 (18)	995 (18)	0.022
60–75 years	5,582 (54)	2,964 (53)	
>75 years	2,914 (28)	1,663 (30)	
Diagnosis			
Osteoarthritis	9,499 (92)	5,165 (92)	0.0018
Other	835 (8.1)	457 (8.1)	
ASA class			
1–2	8,038 (78)	4,296 (76)	0.038
≥3	2,117 (20)	1,242 (22)	
Missing	179 (1.7)	84 (1.5)	
Tranexamic acid			
Yes	9,865 (96)	5,157 (92)	0.17
No	370 (3.6)	417 (7.4)	
Missing	99 (1.0)	48 (0.9)	
Patella resurfaced	671 (6.5)	323 (5.8)	0.031
Prosthesis brand			
NexGen			
CR option – PMMA stemmed tibia	3,286 (32)	933 (17)	0.96
CR option – option stemmed tibia	671 (6.5)	146 (2.6)	
CR flex option – PMMA stemmed tibia	669 (6.5)	45 (0.8)	
CR flex precoat – PMMA stemmed tibia	490 (4.7)	79 (1.4)	
Legion CR Standard – Genesis II tibia	1,075 (10)	594 (11)	
Attune CR – fixed bearing S+ tibia	1,822 (18)	491 (8.7)	
Persona CR – stemmed 5-degree tibia	310 (3.0)	57 (1.0)	
Triathlon			
CR – CS All poly tibia	138 (1.3)	595 (11)	
CR X3 – HXLPE cemented tibia	123 (1.2)	1,459 (26)	
CR CS – X3 HXLPE cemented tibia	477 (4.6)	527 (9.4)	
Other brands	1,273 (12)	696 (12)	
Mean operation time, minutes (SD)	85 (21)	82 (21)	0.14

[a] CR/UC: Cruciate retaining/ultra congruent
[b] Estimated using the reversed Kaplan-Meier method

Table 3. Sensitivity analysis. Kaplan-Meier failure probabilities (1 – KM) for revisions of any cause and cumulative incidence function (CIF) for specific causes of revisions, unadjusted and adjusted HRR and SHR, and instrument variable analysis estimated for cases operated on with or without tourniquet during primary cemented CR/UC[a] TKA with both non-resurfaced and resurfaced patella in Norway 2019–2023

Time	Tourniquet (n = 10,334)	No tourniquet (n = 5,622)
3 years		
Revised due to all causes	193	156
1 – KM % (CI)	2.50 (2.16–2.89)	4.22 (3.58–4.97)
Numbers left at risk, n	3,661	1,599
Revised due to tibial loosening	6	22
CIF % (CI)	0.08 (0.04–0.18)	0.60 (0.38–0.92)
Unadjusted estimates		
HRR due to all causes		
< 3 months	1	1.15 (0.82–1.62)
> 3 months	1	1.67 (1.35–2.06)
SHR tibial loosening	1	8.40 (3.45–20.50)
Adjusted estimates		
HRR [b] due to all causes		
< 3 months	1	1.11 (0.75–1.65)
> 3 months	1	1.32 (0.97–1.80)
SHR [c] tibial loosening	1	5.98 (2.14–16.70)
Instrument variable analysis [d]		
HRR due to all causes		
< 3 months	1	1.28 (0.85–1.92)
> 3 months	1	2.86 (2.11–3.89)

[a] CR/UC: Cruciate retaining/ultra congruent

[b] Hazard rate ratio (HRR) adjusted for age, sex, diagnosis, ASA class, tranexamic acid, prosthesis brand and patellar resurfacing. To fulfil the assumption of proportional hazards, Kaplan-Meier curves were inspected, and period of investigation were split at 3 months.

[c] Sub hazard ratio (SHR) adjusted for age, sex, diagnosis, ASA class, tranexamic acid, prosthesis brand, and patellar resurfacing.

[d] Hospital and year of operation Annual prosperity for using tourniquet were used as instruments to adjust for observed and non-observed confounding factors.

Table 4. Specific revision causes within "Other reasons" for primary cemented or hybrid MS/PS/rotating platform TKA with non-resurfaced and resurfaced patella operated on with or without tourniquet in Norway between 2019 and 2023. Values are count (%)

Specific revision cause	Tourniquet	No tourniquet	Standardized mean difference	P value
Malalignment	15 (11)	11 (8.5)	0.0063	0.6
Polyethylene wear	1 (0.7)	0 (0)	0.010	0.3
Bearing dislocation	4 (2.8)	1 (0.8)	0.011	0.2
Patellar dislocation	6 (4.2)	7 (5.4)	0.015	0.7
Instability	31 (22)	25 (19)	0.021	0.6
Periprosthetic fracture	3 (2.1)	13 (10)	0.041	0.006
OA progression	13 (9.1)	10 (7.7)	0.0020	0.7
Stiffness	26 (18)	26 (20)	0.025	0.7
Pain only	14 (9.8)	14 (11)	0.020	0.8
Other	30 (21)	23 (18)	0.0068	0.5
Missing	1 (0.7)	2 (1.5)	0.013	0.3
Total	144	132	0.044	

Table 5. Sensitivity analysis. Kaplan-Meier cumulative mortality rate (1 – KM) for patients operated on with or without tourniquet during their primary cemented CR/UC TKA with both non-resurfaced and resurfaced patella in Norway between 2019 and 2023. Cox adjusted hazard rate ratio (HRR) for risk of death

Time	Tourniquet (n = 9,289)	No tourniquet (n = 5,061)
30 days		
Death, n	3	3
1 – KM % (CI)	0.03 (0.01–0.19)	0.06 (0.02–0.19)
Numbers left at risk, n	9,089	4,927
1 year		
Death, n	35	26
1 – KM % (CI)	0.48 (0.32–0.62)	0.62 (0.42–0.91)
Numbers left at risk, n	6,891	3,189
3 years		
Death, n	126	82
1 – KM % (CI)	2.34 (1.96–2.80)	3.14 (2.51–3.93)
Numbers left at risk, n	3,231	1,425
Unadjusted estimates		
HRR (CI)	1	1.27 (1.00–1.60)
Adjusted estimates		
HRR [a] (CI)	1	1.27 (0.99–1.63)

[a] HRR no tourniquet vs with tourniquet (reference). Adjusted for age, sex, diagnosis, ASA class, and tranexamic acid.

Table 6. Sensitivity analysis with single missing imputation, presenting adjusted Cox regression and adjusted Fine and Gray analysis with “best case” and “worst case” scenario for tranexamic acid and ASA class on cases being operated on with or without tourniquet during primary cemented CR/UC TKA both non-resurfaced and resurfaced patella in Norway 2019–2023

	Tourniquet (reference)	No tourniquet
“Best case” [a]		
HRR [c] all causes		
< 3 months	1	1.07 (0.73–1.58)
> 3 months	1	1.33 (0.98–1.80)
SHR [d] tibial loosening	1	5.91 (2.12–16.5)
“Worst case” [b]		
HRR [c] all causes		
< 3 months	1	1.07 (0.73–1.58)
> 3 months	1	1.33 (0.98–1.81)
SHR [d] tibial loosening	1	5.96 (2.14–16.6)

[a] Best case: Tranexamic acid and ASA class 1 and 2

[b] Worst case: No tranexamic acid and ASA class 3 and 4

[c] Hazard rate ratio (HRR) adjusted for age, sex, diagnosis, ASA class, tranexamic acid, patella component and implant brand. To fulfill the proportional hazards assumption Kaplan-Meier curves were inspected and period of investigation were split at 3 months.

[d] Sub-hazard ratio (SHR) adjusted for age, sex, diagnosis, ASA class, tranexamic acid, patella component and implant brand.

Figures

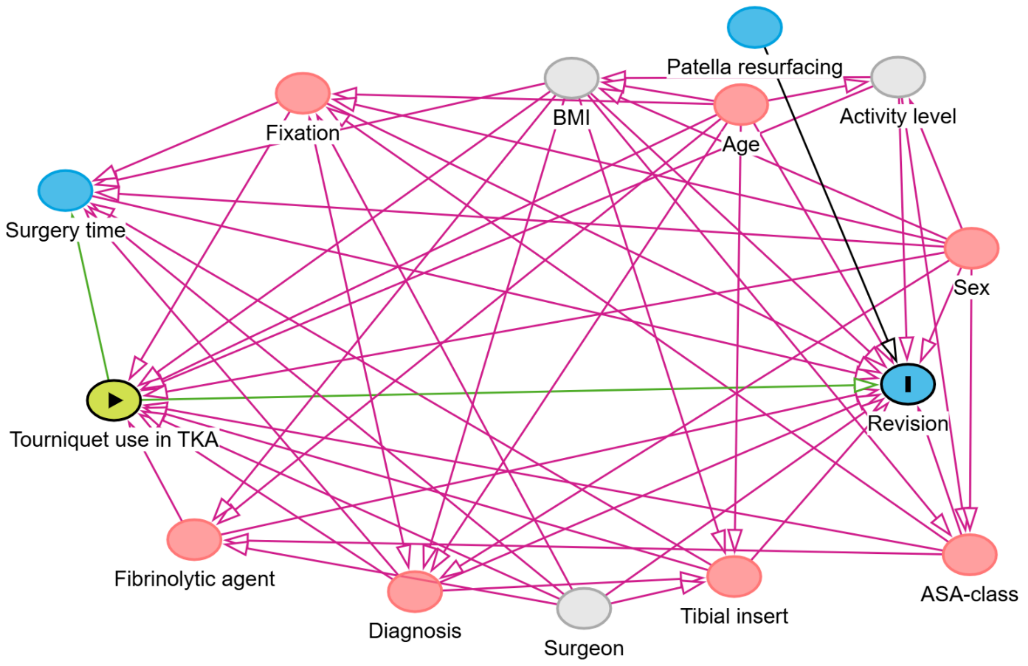

Figure A. DAG illustrating potential confounders and factors adjusted for in Cox regression and Fine & Gray models. Tourniquet use (green node) is the exposure; implant failure (blue node) is the outcome. The green arrow represents the causal pathway of interest. Red nodes and arrows represent potential confounders. Surgery time is considered a potential mediator. BMI, activity level, and surgeon were unmeasured and therefore not adjusted for; see the main text for details.

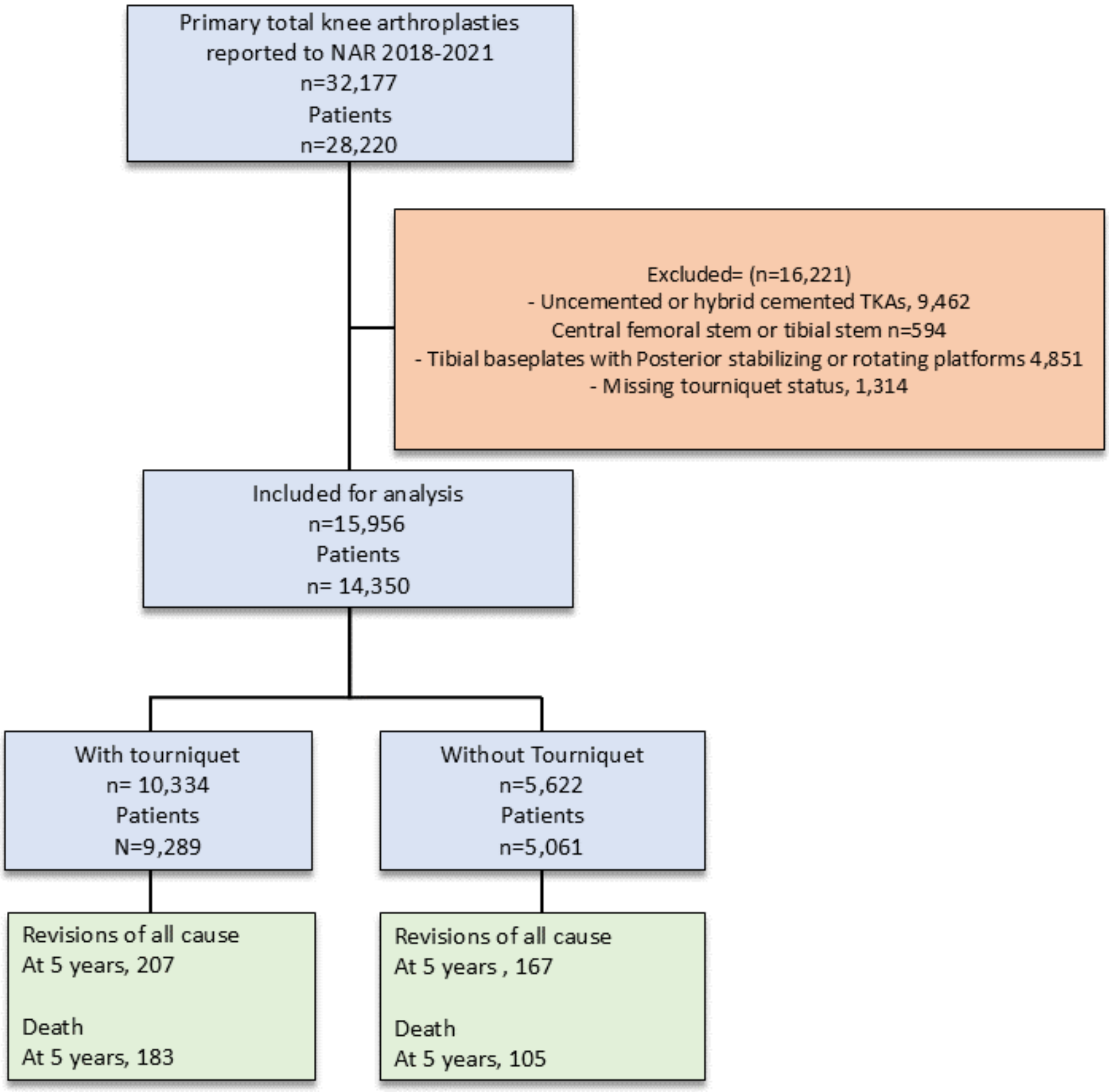

Figure B. Demographic flowchart for sensitivity analysis
